# Nonlinear Nanoscale Electrical Transport Distinguishes Fibroblasts and Osteoblasts

**DOI:** 10.1021/acsomega.6c02152

**Published:** 2026-06-15

**Authors:** Erick Bolaños Torres, Rosemayre Freire, Brandon Sousa, Francisco Carlos Carneiro Soares Salomão, Eduardo Barros, Jeanlex de Sousa, Claudio Lucas Nunes de Oliveira

**Affiliations:** † Departamento de Física, 28121 Universidade Federal do Ceará, Fortaleza, Ceará 60020-181, Brazil; ‡ Central Analítica, Universidade Federal do Ceará, Fortaleza, Ceará 60455-760, Brazil; § Departamento de Bioquímica e Biologia Molecular, 28121 Universidade Federal do Ceará, Fortaleza, Ceará 60.4351-970, Brazil; ∥ 67843 Universidade Estadual do Ceará, Faculdade de Filosofia Dom Aureliano Matos, Limoeiro do Norte, Ceará 60740-000, Brazil

## Abstract

Electrical signaling plays a central role in cellular function, yet direct mapping of electrical transport within individual cells at the nanoscale remains challenging. Conventional electrophysiological and impedance-based techniques either lack sufficient spatial resolution or significantly perturb cellular properties. Here, we employ conductive atomic force microscopy (CAFM) to probe effective local current–voltage (I–V) characteristics with subcellular resolution in fixed and air-dried cells from two mammalian cell types: L929 fibroblasts and OFCOLII osteoblasts cultured on conductive indium tin oxide (ITO) substrates. Spatially resolved current maps reveal a systematically enhanced electrical response in nuclear regions compared to the cytoplasmic periphery in both cell types. L929 fibroblasts exhibit predominantly linear, ohmic-like I–V behavior, whereas OFCOLII osteoblasts display pronounced nonlinear, diode-like electrical transport. Quantitative analysis reveals strong cell-type- and region-dependent variations in effective resistance and capacitance, highlighting the role of intracellular organization in shaping nanoscale electrical behavior. These results establish CAFM as a powerful approach for resolving subcellular electrical heterogeneity and demonstrate that distinct electrical transport signatures can differentiate cellular phenotypes at the single-cell level. These findings position nanoscale electrical transport as a physically grounded marker of cellular phenotype.

## Graphical abstract

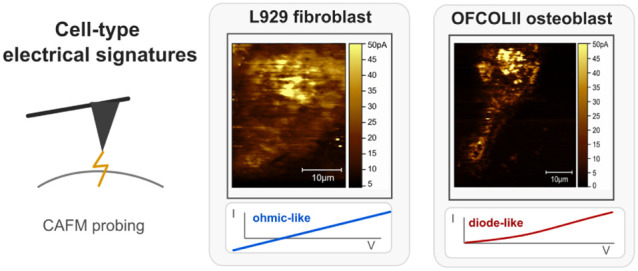

## Introduction

Electrical transport within cells provides a sensitive window into cellular physiological state and biochemical activity, capturing functional changes that may not be accessible through purely mechanical or morphological analyses.^1^ Cells comprise a complex and heterogeneous assembly of biomolecules, membranes, cytoskeletal filaments, and nucleic acids, each contributing to cellular function and organization.^2^ Variations in their electrical properties have been linked to pathological processes including cardiovascular disorders, altered cell motility, and cancer.^3^ A quantitative understanding of charge transport at the cellular and subcellular levels could therefore inform new strategies for disease diagnosis, therapeutic monitoring, and drug development.^4,5^

A major challenge in probing cellular electrical behavior is achieving high sensitivity while minimizing perturbation of the native structure. The *patch-clamp* technique, pioneered by Neher and Sakmann, revolutionized electrophysiology by enabling direct measurements of ionic currents through individual channels.^6^ Despite its unparalleled temporal resolution, patch-clamp methods are inherently low-throughput, experimentally demanding, and limited in spatial resolution.^7^ Complementary approaches, including fluorescence-based techniques, electrical impedance spectroscopy, and other single-cell electrical characterization methods, provide indirect or spatially averaged access to cellular electrical properties.^8–10^ However, fluorescence imaging typically requires exogenous dyes that may introduce phototoxicity or perturb cellular function,^11^ while impedance spectroscopy is constrained by electrode–cell coupling, averaging effects, and contributions from the extracellular medium.^12–19^ These limitations motivate the development of alternative methodologies capable of mapping electrical properties at the nanoscale with minimal structural disruption.

Recent AFM-based studies have shown that nanoscale mechanical and structural mapping can resolve subcellular heterogeneity^20,21^ while electrostatic, dielectric, and conductive measurements can reveal biomolecular organization and cell–material interfacial effects that are not accessible through conventional ensemble electrical techniques.^22–25^ In the context of osteogenic systems, biomineralized collagen matrices and ion-rich extracellular environments can further influence local dielectric and transport properties, motivating nanoscale electrical measurements in osteoblast-like cells.^26^

Atomic force microscopy (AFM) has emerged as a versatile platform for nanoscale biophysical characterization, enabling simultaneous measurements of topography and mechanical properties such as stiffness and viscoelasticity.^27–30^ In its electrical modes, such as conductive AFM (CAFM), Kelvin probe force microscopy (KPFM), and electrostatic force microscopy (EFM), AFM further enables the quantification of local current, electrostatic forces, and surface potential with nanometer-scale spatial resolution.^31^ CAFM, in particular, has been extensively applied to investigate charge-transport phenomena in biological systems ranging from proteins and DNA to complex biomaterials.^32,33^ These studies have shown that electrical transport in biological matter depends sensitively on molecular orientation, hydration state, and measurement environment. Moreover, CAFM has revealed nontrivial electronic behavior in biogenic nanostructures such as neuromelanin and ferritin,^34^ as well as cell-type-dependent electrical signatures in neurons, astrocytes, and cancer cells.^35–37^ Together, these findings underscore CAFM’s potential for linking nanoscale charge transport to biological structure and function.

Despite these advances, the use of CAFM as a quantitative tool for probing electrical transport in intact cells and at cell–material interfaces remains comparatively underexplored. Understanding how different cell types conduct charge at the nanoscale is particularly relevant for the design of electroactive biomaterials, implant coatings, and bioelectronic devices, where cellular adhesion, differentiation, and functional state are strongly influenced by electrical and electrochemical interactions at conductive interfaces. Fibroblasts and osteoblasts play central roles in tissue repair, regeneration, and remodeling, making them compelling model systems for investigating how intrinsic cellular composition and organization influence nanoscale electrical transport, particularly when assessed under strictly controlled and comparable experimental conditions.

Unlike previous CAFM studies focusing on isolated biomolecules or single cell types, the present work provides a direct, statistically supported comparison of subcellular electrical transport across two distinct mammalian lineages under identical experimental conditions. Specifically, we apply CAFM to systematically investigate spatially resolved electrical transport in L929 fibroblasts and OFCOLII osteoblasts cultured on conductive substrates. Beyond descriptive mapping, the present work demonstrates that nonlinear electrical response can serve as a distinguishing nanoscale signature of cellular phenotype. By correlating confocal fluorescence microscopy, nanoscale topography, and local current–voltage spectroscopy, we identify distinct, cell-type-dependent electrical responses at the subcellular level. We show that fibroblasts exhibit predominantly linear, ohmic-like behavior, whereas osteoblasts display pronounced nonlinear, diode-like current–voltage characteristics indicative of voltage-dependent transport mechanisms. These results demonstrate that CAFM can resolve subcellular electrical heterogeneity with high spatial resolution and reveal fundamentally different charge-transport behaviors arising from the structural and compositional organization of different cell types.

## Methods

### Cell Culture

L929 fibroblasts (BCRJ code 0188) and OFCOLII osteoblast cells (BCRJ code 0192), obtained from the Rio de Janeiro Cell Bank (BCRJ), were cultured on indium tin oxide (ITO)-coated glass slides. Prior to cell seeding, the substrates were thoroughly cleaned, sterilized, and pretreated with a 0.1% poly-l-lysine (PLL) solution. The PLL was left in contact with the surface for 15 min, after which the slides were washed with phosphate-buffered saline (PBS) and allowed to air-dry. Cells were seeded at a density of 1.25 × 10^5^ cells cm^–2^ and incubated for 24 h following a previously established protocol.^38^ Cell culture was performed in Dulbecco’s Modified Eagle Medium (DMEM, high glucose), supplemented with 10% fetal bovine serum and 1% penicillin-streptomycin, and maintained at 37 °C in a humidified atmosphere containing 5% CO_2_.

### Confocal Microscopy

Cells were fixed with paraformaldehyde and permeabilized using Triton X-100 prior to staining. Nonspecific binding sites were blocked with bovine serum albumin (BSA). F-actin filaments were stained using phalloidin, and cell nuclei were counterstained with 4′,6-diamidino-2-phenylindole (DAPI, 100 ng mL^–1^ in PBS). Confocal fluorescence images were acquired at room temperature using a Zeiss LSM 710 laser-scanning confocal microscope (Zeiss, Jena, Germany). Excitation wavelengths of 405 and 488 nm were used, with corresponding emission maxima at 457 nm for DAPI and 518 nm for phalloidin-labeled F-actin.

### Conductive AFM Measurements

Conductive atomic force microscopy (CAFM) measurements were carried out using an MFP-3D-BIO AFM system integrated with an inverted optical microscope (Asylum Research, Santa Barbara, CA). Measurements were performed in Optimized Resistance Conductance Amplifier (ORCA) mode, which is specifically designed for conductive contact-mode operation. The standard ORCA sample holder was modified to accommodate conductive ITO substrates, enabling both optical alignment and electrical measurements. A conductive magnet was attached to the substrate to establish a closed electrical circuit, with the AFM tip serving as the source electrode and the ITO substrate as the common ground for all cells. During scanning, current–voltage (I–V) data were acquired point-by-point to generate spatially resolved effective electrical response maps.

Conductive Pt-coated CONTPt probes (NanoWorld) with a nominal spring constant of *k* = 0.2 N m^–1^ and a resonance frequency of *f* = 14 kHz were used throughout the study. The cantilever spring constant was calibrated prior to each experiment using the thermal noise method. Control I–V measurements were performed on the bare ITO substrate before and after each experiment to verify tip integrity, baseline conductivity, and instrumental stability. Repeated I–V measurements were performed at selected subcellular locations to assess reproducibility and evaluate possible measurement-induced modifications of the sample. Four consecutive sets of I–V curves were acquired at the same locations, and the electrical response remained stable over repeated acquisitions, with no significant degradation or drift in the current signal, as confirmed by the repeated measurements shown in Figures 1SM and 2SM of the Supporting Information.

To minimize artifacts and ensure reproducibility, CAFM measurements were performed on fixed, nonviable, and air-dried cells, not on living cells. Under these conditions, the cells are not physiologically active, and active processes such as membrane-potential regulation, Ca^2+^-mediated ion-channel activity, and transmembrane ionic currents are suppressed. Fixation was employed to provide mechanically stable samples suitable for high-resolution current mapping under well-defined tip–sample contact conditions, while minimizing contributions from ionic mobility, hydration-dependent conduction, and electrochemical reactions. After 24 h of culture, cells were removed from the incubator, rinsed three times with phosphate-buffered saline (PBS, 5 min each), fixed with 4% paraformaldehyde for 15 min, and rinsed twice with deionized water to remove residual salts, culture-medium components, and weakly bound chemical residues. The samples were then air-dried prior to CAFM measurements, and the conductive magnet was affixed to the substrate. Therefore, the measured currents should be interpreted as an effective electrical response of the fixed cellular structure rather than as active physiological electrical signaling.

Current maps were acquired using a maximum normal force of 5.6 nN at a scan rate of 0.2 Hz. A bias voltage of 5 V was applied to ensure stable electrical contact between the AFM tip and the cell without inducing mechanical damage. Given that the AFM tip indentation depth under these conditions is negligible compared to the overall cell thickness, the structural integrity of the samples was preserved throughout the measurements. In cases where electrical contact was insufficient, the maximum applied force was increased up to 10 nN, a threshold previously shown to minimize cellular damage under conductive AFM measurements.^39^

Topographic and current images were processed using Gwyddion software (version 2.65).^40^ For detailed electrical characterization, I–V curves were acquired at selected, well-defined subcellular locations using a voltage sweep from −10 V to +10 V at a frequency of 0.1 Hz. Parasitic capacitance contributions were evaluated following the framework described in ref 41. The measured CAFM current may contain both conductive current through the sample and displacement currents associated with parasitic capacitances from the cantilever, probe holder, substrate, wiring, and surrounding setup. To estimate this background, control measurements were first performed with the AFM tip slightly retracted from the sample surface. Under these conditions, the direct conductive pathway through the sample is suppressed, and the measured signal is dominated by capacitive and instrumental contributions.

In addition, bidirectional I–V sweeps were analyzed by noting that the displacement current is proportional to the voltage sweep rate and therefore changes sign between forward and backward sweeps. Thus, the average of the forward and backward branches provides the conductive component, whereas their difference provides the capacitive contribution. This procedure improves the reliability of the extracted effective electrical parameters.

## Results and Discussion

### Morphology of L929 and OFCOLII Cells by Confocal Microscopy

Figure 1a and b show confocal fluorescence microscopy images of representative L929 fibroblast and OFCOLII osteoblast cells, respectively. In both cell types, F-actin filaments (green) form an extended filamentous network spanning the cell body, with pronounced enrichment toward the cell periphery. Cell nuclei (blue) are centrally located in both cases, consistent with the morphology of adherent cells.

**Figure 1:**
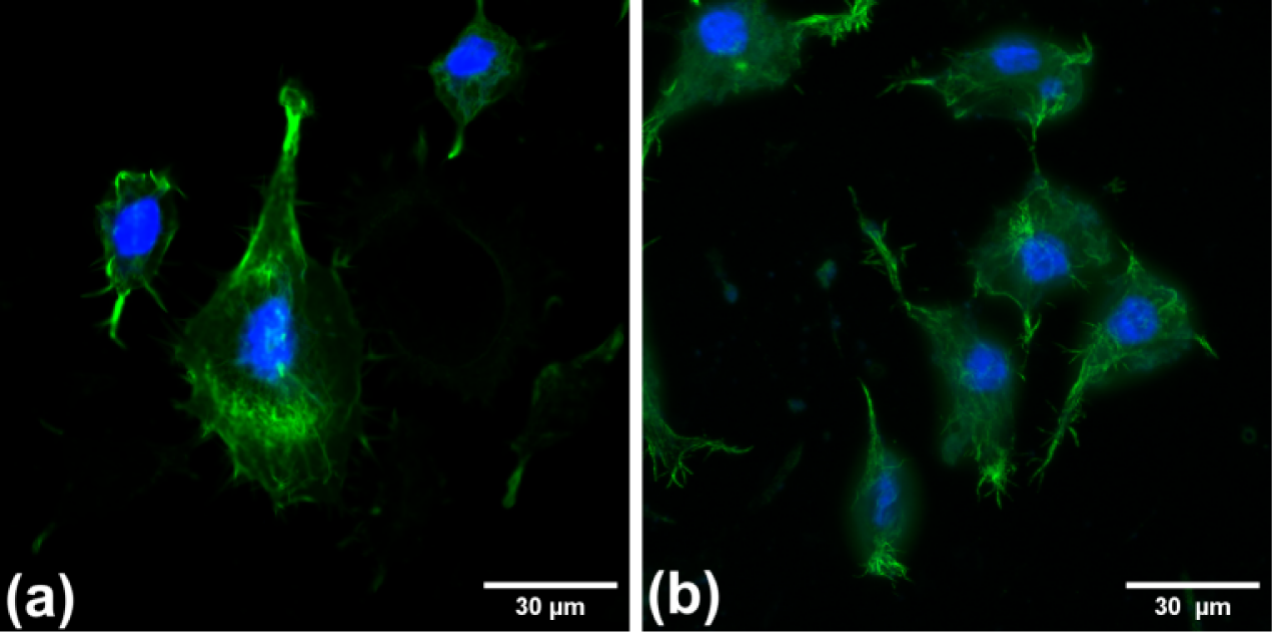
Fluorescence microscopy images of L929 (a) and OFCOLII (b) cells showing actin cytoskeletal organization. Actin filaments (green) exhibit pronounced peripheral enrichment and intracellular filamentous structures, while nuclei (blue) are centrally localized. The images illustrate distinct cellular morphologies and differences in cell spreading and actin organization between the two cell types. Scale bars: 30 μm.

Despite these common features, clear morphological differences are observed between the two lineages. L929 cells exhibit a relatively spread morphology, characterized by short peripheral extensions and a flattened nuclear region. In contrast, OFCOLII cells display more elongated protrusions and a distinctly rounded nuclear morphology, reflecting of differences in cytoskeletal organization and overall cell geometry. These variations reflect cell-type-specific structural adaptations to adhesion on the ITO-coated substrate.

Importantly, both cell types exhibit robust adhesion and spreading on the conductive ITO surface, demonstrating the substrate’s biocompatibility and suitability for nanoscale electrical measurements. This observation is consistent with previous reports on cell–ITO interfaces.^42,43^ Moreover, despite the collapse in cell height due to intracellular water loss, the preserved organization of the actin cytoskeleton and the well-defined nuclear morphology indicate that the fixation protocol maintained cellular structural integrity, providing a reliable morphological basis for the subsequent CAFM current mapping and current–voltage measurements. As discussed above, the measured currents reflect an effective electrical response influenced by local geometry and molecular composition rather than intrinsic bulk conductivity.

### Electrical Conductivity in L929 Cells

The topographic and current maps acquired for a representative L929 fibroblast cell are shown in Figure 2a and b, respectively. The topographic image reveals a pronounced protrusion located in the central region of the cell body. Comparison with confocal fluorescence microscopy indicates that this elevated region corresponds to the cell nucleus (green rectangle in Figure 2a), while the region highlighted in blue identifies the actin-rich peripheral cytoplasm. The corresponding current map, recorded under an applied bias of 5 V, shows systematically higher current levels in the nuclear region than in the cell periphery, indicating a spatially heterogeneous electrical response across the cell.

**Figure 2:**
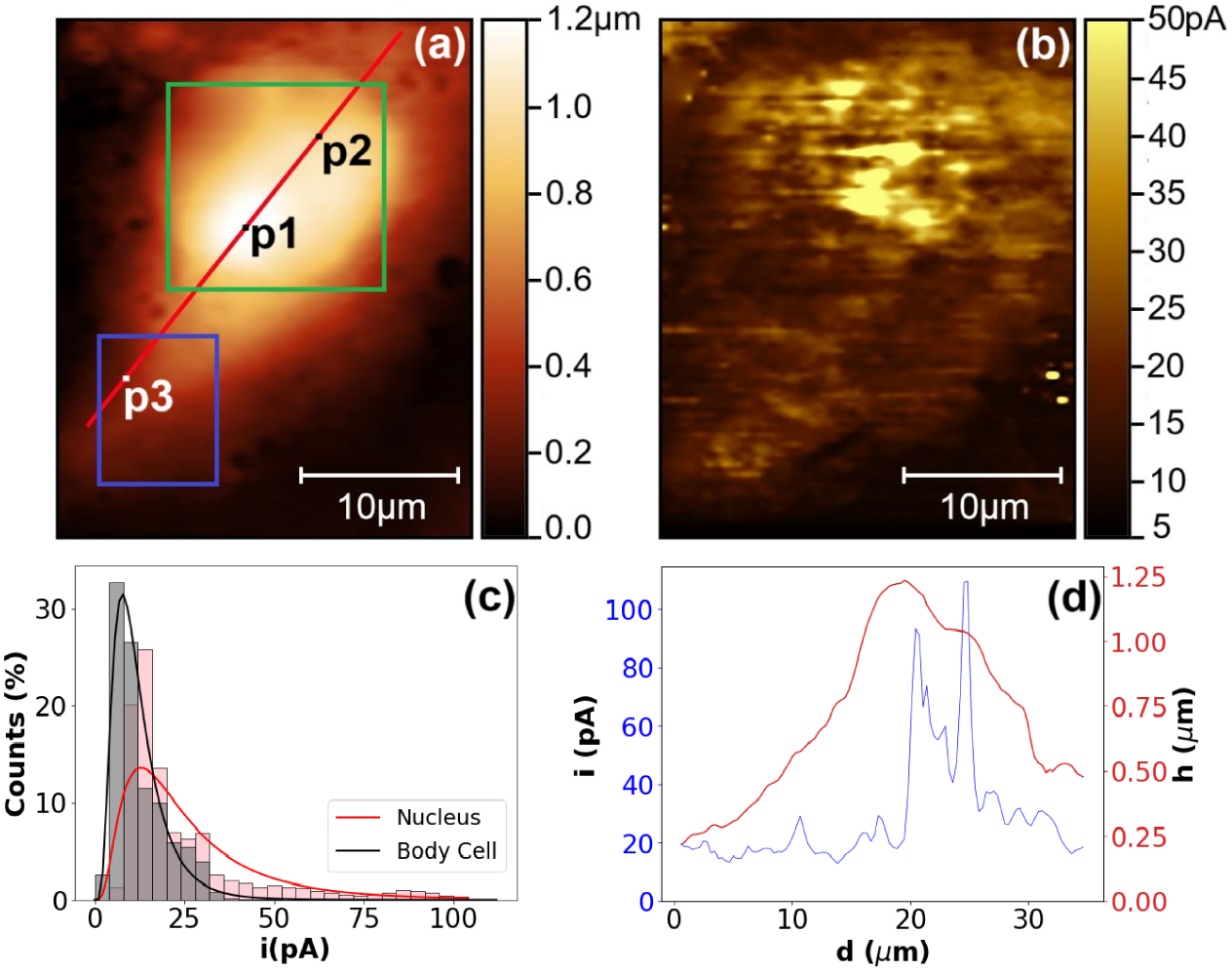
Conductive atomic force microscopy mapping of a representative L929 fibroblast cell. (a) Topographic image and (b) corresponding current map acquired under an applied bias of 5 V. The topography reveals the characteristic cell morphology, while the current map shows spatially heterogeneous electrical response across the cell. (c) Distributions of the measured current in the nuclear (red) and peripheral (black) regions. The peripheral distribution exhibits a taller peak because many pixels are concentrated in a narrow low-current range, whereas the nuclear distribution is broader and shifted toward higher current values, indicating that higher current values are more frequent in the nuclear region. The modal current values are 13.1 pA for the nuclear region and 5.82 pA for the peripheral region. (d) Line profiles of height (red) and current (blue) extracted along the red line in (a) demonstrate a clear correlation between increased topographic height and enhanced electrical current in the nuclear region relative to the cell periphery.

Cell dimensions were quantified as length (distance between the opposite extremities), width (maximum orthogonal distance), and height (maximum distance from the substrate). The average length and width were 34.56 ± 1.31 μm and 23.50 ± 0.87 μm, respectively (*n* = 12), in good agreement with reported values for L929 fibroblasts.^44^ The mean cell height was 894 ± 57 nm (*n* = 12).

Within the nuclear region, two distinct subregions can be identified: a higher, rounded area (point 1) and a lower, flatter region (point 2), as indicated in Figure 2a. Figure 2d presents the height and current profiles extracted along the red line in Figure 2a. Point 1 corresponds to the maximum elevation and is associated with the nucleolar region, while point 2 corresponds to the surrounding nuclear envelope. Point 3, located at the actin-rich cell periphery, lies outside the nuclear region and exhibits both reduced height and lower measured current. The close correspondence between the height (red) and current (blue) profiles indicates that regions of increased topographic elevation are associated with enhanced current levels, consistent with previous CAFM observations on cellular systems.^45^

Figure 2c compares the current distributions extracted from the nuclear and peripheral regions. The peripheral distribution exhibits a taller, narrower peak because most pixels in this region are concentrated within a low-current interval, mainly in the 4–10 pA range. In contrast, the nuclear distribution is broader and shifted toward higher current values, with currents above 10 pA being more frequent in the nuclear region. Thus, the histogram supports the current map in Figure 2b: the peripheral region contains many low-current pixels, whereas the nuclear region displays higher current values over a broader range. Consistently, the average current in the nuclear region was 28.7 ± 5.7 pA (*n* = 12), approximately 70% higher than the peripheral value of 17.0 ± 1.8 pA (*n* = 12). Additional 2 × 2 μm^2^ CAFM maps acquired in selected cell body/peripheral and nuclear regions of L929 cells are provided in Figure 3SM of the Supporting Information, further illustrating the local topographic and current heterogeneity at the subcellular scale.

The higher current observed in the nuclear region compared with the cytoplasmic periphery is attributed to a combination of local geometry, tip–sample contact, and intracellular molecular organization. Importantly, the topographic effect acts against a purely thickness-based interpretation: the nuclear region is thicker than the peripheral region but exhibits lower effective resistance and higher current. In a simple vertical transport picture, a larger thickness would be expected to increase the effective path length and, therefore, the resistance. The observed trend, therefore, indicates that thickness alone cannot account for the enhanced nuclear current. Instead, the nucleus contains a dense assembly of nucleic acids, nucleoprotein complexes, chromatin-associated structures, and other biomolecular components that can modify the local dielectric environment, polarization response, charge-storage behavior, and effective charge-transport pathways. In contrast, the actin-rich peripheral region forms a structurally distinct network that can exhibit a higher effective resistance under CAFM conditions. Therefore, the measured current should be interpreted as an effective nanoscale electrical response governed by morphology, tip–sample contact, and molecular composition, rather than as a purely geometrical effect.

Current–voltage (I–V) measurements were performed at points 1–3 (Figure 3) using a voltage sweep from −10 V to +10 V at a frequency of 0.1 Hz. The resulting I–V curves exhibit approximately linear current–voltage dependence, with only slight hysteresis between forward and reverse sweeps. This quasi-linear response indicates an effective ohmic-like behavior, with relatively weak interfacial barriers and minor capacitive contributions. The small hysteresis observed in L929 cells is consistent with reversible interfacial polarization and limited charge storage at the tip–cell–ITO contact. Accordingly, the electrical response of L929 cells can be described phenomenologically by an effective resistor in parallel with a constant capacitance.

**Figure 3:**
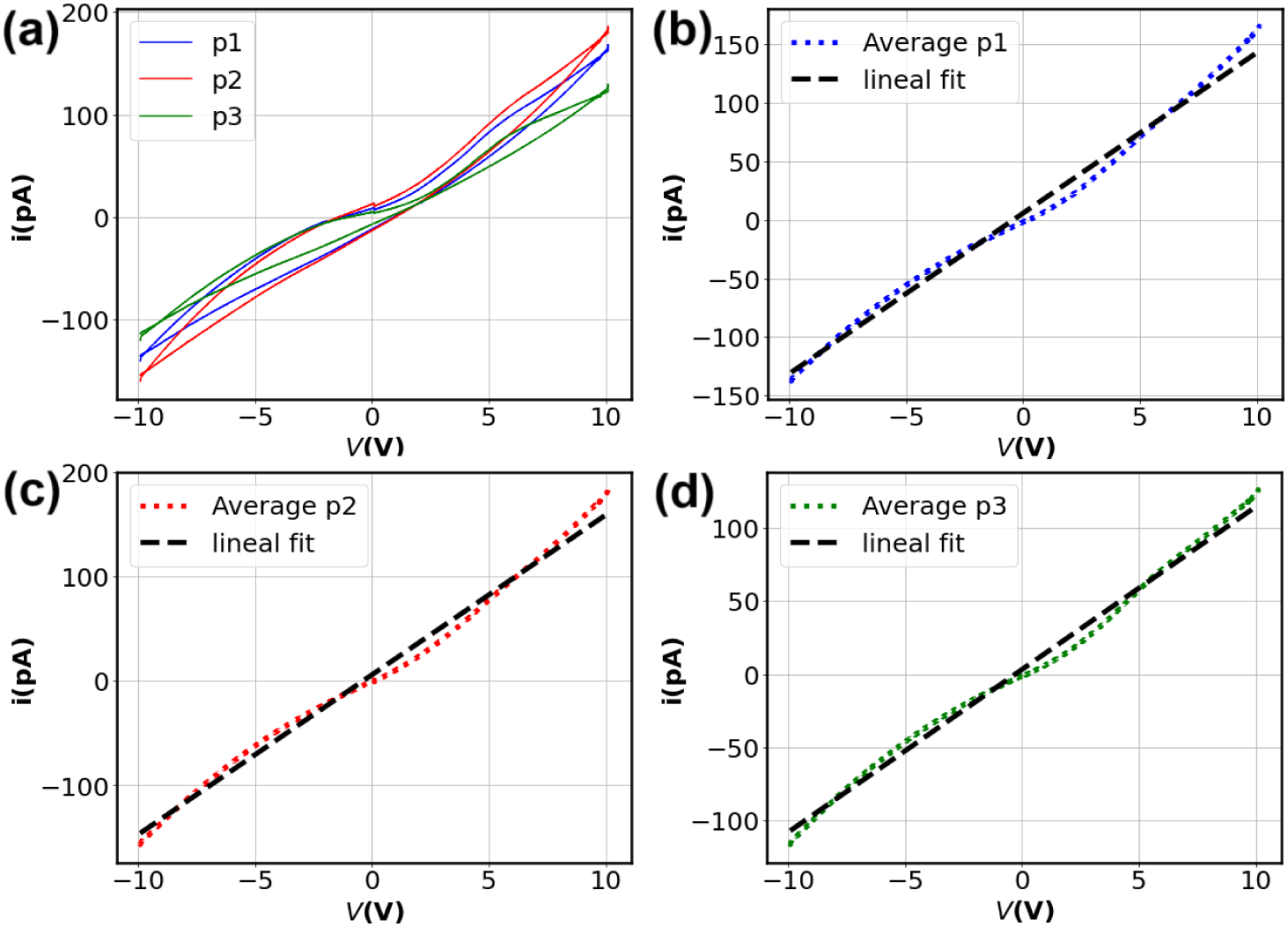
Current–voltage (I–V) characterization of an L929 fibroblast cell measured by CAFM. (a) Individual I–V curves acquired at the three locations indicated in Figure 2a (p1–p3) under a voltage sweep from −10 V to +10 V. (b–d) Averaged I–V responses at p1 (nucleolus), p2 (nucleus), and p3 (cell periphery), respectively, together with linear fits (black dashed lines). The different cellular regions exhibit distinct electrical responses, including variations in slope and deviations from ideal linearity, suggesting region-dependent charge transport and storage characteristics.

For quantitative analysis, four I–V curves were acquired at each measurement point, and their averages were fitted using a linear model, as shown in Figure 3b–d. Only curves with a correlation coefficient *R* ^2^ > 0.97 were included in the statistical analysis. The resulting resistance distributions, summarized in Figure 4a, confirm that the nuclear region exhibits a lower effective resistance than the cell periphery. Repeated I–V measurements confirmed that the approximately ohmic response of L929 fibroblasts was reproducible and did not arise from progressive tip-induced sample degradation or signal drift (Figure 1SM).

**Figure 4:**
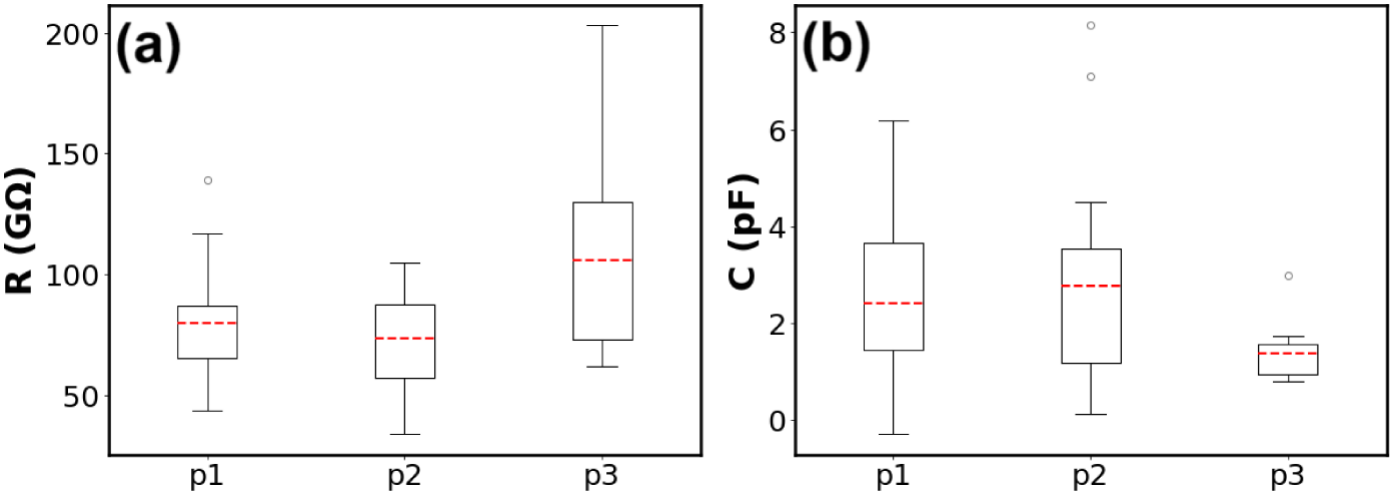
Statistical distributions of electrical parameters extracted from CAFM measurements on L929 fibroblast cells. (a) Resistance and (b) capacitance obtained at the three locations p1–p3 (number of measurements: *n*_*p*1_ = 12, *n*_*p*2_ = 11, *n*_*p*3_ = 11). Boxes represent the interquartile range (Q1–Q3, 25–75% of the data), whiskers extend to Q1 – 1.5 × IQR and Q3 + 1.5 × IQR, red dashed lines indicate mean values, and circles denote outliers.

Although the L929 I–V curves are predominantly linear, a slight deviation from ideal ohmic behavior is observed in the low-bias region, particularly around 1–2 V. This feature is interpreted as a low-bias nonideality associated with interfacial effects, including weak capacitive contributions, local polarization, and nonideal tip–sample electrical contact. To evaluate possible bias-dependent asymmetry, the positive- and negative-bias branches were also fitted separately. The extracted slopes were comparable and of the same order of magnitude, although a moderate asymmetry between both polarities was observed. The slope obtained from the global linear fit lies within the range defined by the positive- and negative-bias fits. Therefore, the global fit was retained as a single effective resistance parameter for statistical comparison across subcellular regions and cells.

In CAFM, the electric field is highly nonuniform because of the tip–plane geometry and is concentrated near the conductive AFM tip apex. Therefore, the resistance extracted from the I–V slope should be interpreted as an effective CAFM resistance rather than as a bulk material resistance or intrinsic resistivity. This effective parameter includes contributions from the local field distribution, tip–sample contact area, interfacial effects, local cell thickness, and the heterogeneous cellular structure. The approximation used here is not that the electric field is uniform, but that, under a fixed CAFM geometry and identical experimental protocol, the slope of an approximately linear I–V curve provides a consistent phenomenological descriptor for comparing different subcellular regions and, when applicable, different cell types measured under the same protocol.

The mean electrical resistance extracted for the nucleolar region (p1) was 79.64 GΩ, slightly higher than that of the surrounding nuclear region (p2, 73.74 GΩ). In contrast, the peripheral region (p3) exhibited a significantly higher resistance of 105.72 GΩ. These differences demonstrate that the internal organization of the L929 cell gives rise to spatially heterogeneous electrical responses at the nanoscale. The resistance values reported here are one to 2 orders of magnitude higher than those typically measured for fibroblast membranes^46,47^ reflecting the increased impedance associated with fixed samples and the dry contact conditions inherent to CAFM measurements.

To estimate the local capacitance, the electrical response was modeled using a parallel RC circuit. The resistance of the underlying ITO substrate, which is connected in series with the cell, is at least 5 orders of magnitude smaller than the measured cellular resistance and can therefore be neglected. Contributions from displacement currents associated with the capacitance between the cantilever and the substrate were corrected following the procedure described in ref 41.

The forward (*i*_forward_) and backward (*i*_backward_) currents can be expressed as

1 $$i_{\mathrm{forward}}=\frac{V}{R}+C\nu$$

2 $$i_{\mathrm{backward}}=\frac{V}{R}-C\nu$$

where *V* is the applied voltage, *R* is the resistance, *C* is the capacitance, and ν is the voltage sweep rate. From these expressions, the capacitance is obtained as

3 $$C=\frac{\Delta i}{2\nu }$$

with Δ*i* = *i*_backward_ – *i*_forward_.

The resulting capacitance distributions are shown in Figure 4b. The mean capacitance values for the nucleolar (p1) and nuclear (p2) regions were 2.40 pF and 2.73 pF, respectively, and were statistically similar within experimental uncertainty. In contrast, the peripheral region (p3) exhibits a reduced mean capacitance of 1.37 pF. These values are slightly lower than those reported for living cardiac fibroblasts (4.5 ± 0.4 pF),^48^ consistent with the reduced ionic mobility and altered dielectric environment in fixed cells. Overall, L929 fibroblast cells display high effective resistance and low capacitance, indicative of limited charge transport and modest charge storage capacity under the present experimental conditions.

### Electrical Conductivity in OFCOLII Cells

The experimental configuration and CAFM parameters employed for OFCOLII osteoblast cells were identical to those used for L929 fibroblasts, allowing direct comparison between the two cell types. The average length and width of OFCOLII cells were 44.3 ± 1.9 μm and 25.3 ± 0.9 μm, respectively (*n* = 12), in agreement with reported dimensions for osteoblasts.^49^ The mean cell height was 720 ± 61 nm (*n* = 12), slightly lower than that measured for L929 cells.

Comparison of the CAFM topography with fluorescence microscopy images indicates that the highest region of the OFCOLII cell corresponds to the nuclear area (green rectangle in Figure 5a), while the peripheral region (blue) is enriched in actin filaments (Figure 1b). The corresponding current map acquired under a 5 V applied bias (Figure 5b) exhibits enhanced current levels in the nuclear region relative to the cell periphery, indicating a spatially heterogeneous electrical response similar to that observed for L929 cells.

**Figure 5:**
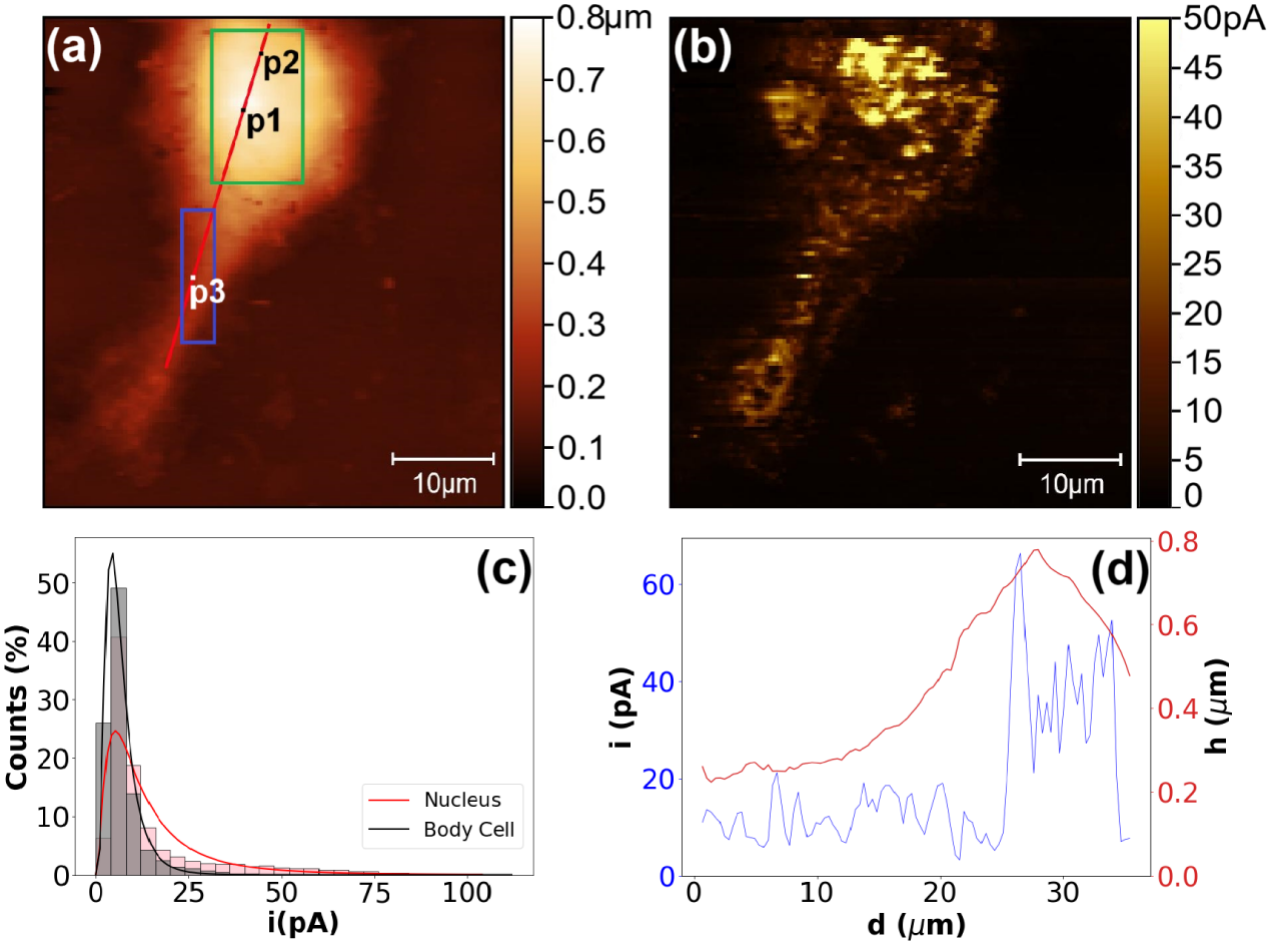
Conductive atomic force microscopy mapping of a representative OFCOLII osteoblast cell. (a) Topographic image and (b) corresponding current map acquired under an applied bias of 5 V. (c) Distributions of the measured current in the nuclear and peripheral regions reveal distinct conductive responses between central and peripheral cellular compartments. (d) Height (red) and current (blue) line profiles extracted along the red line in (a) show enhanced current levels in the nuclear region relative to the cell periphery. Scale bars: 10 μm.

As with the L929 cells, within the nuclear region, a rounded high-elevation zone corresponding to the nucleolus (p1) and a flatter surrounding nuclear region (p2) were identified (Figure 5a). Height and current profiles extracted along the red line in (Figure 5a, d) reveal a clear correspondence between local topography and measured current, with higher current levels observed in regions of greater height. Current distributions (Figure 5c) yield mean current values of 21.9 ± 4.8 pA in the nuclear region and 7.9 ± 0.9 pA at the cell periphery (*n* = 12), reproducing the same qualitative trend observed for L929 fibroblasts, albeit with lower absolute current amplitudes. Additional 2 × 2 μm^2^ CAFM maps acquired in selected cell body/peripheral and nuclear regions of OFCOLII cells are provided in Figure 4SM of the Supporting Information, showing local topographic and current heterogeneity at higher spatial detail.

Figure 6a presents the I–V curves recorded at points 1–3 using voltage sweeps from −10 V to +10 V at 0.1 Hz. In contrast to the approximately linear response observed for L929 cells, OFCOLII osteoblasts exhibit pronounced nonlinear current–voltage characteristics. The forward and reverse voltage sweeps follow distinct trajectories, particularly at higher bias voltages, indicating voltage-dependent electrical behavior and significant capacitive and nonlinear transport contributions. Repeated I–V measurements further confirmed that the nonlinear and hysteretic response of OFCOLII osteoblasts was reproducible over consecutive acquisitions and was not caused by progressive sample degradation during CAFM measurements (Figure 2SM).

**Figure 6:**
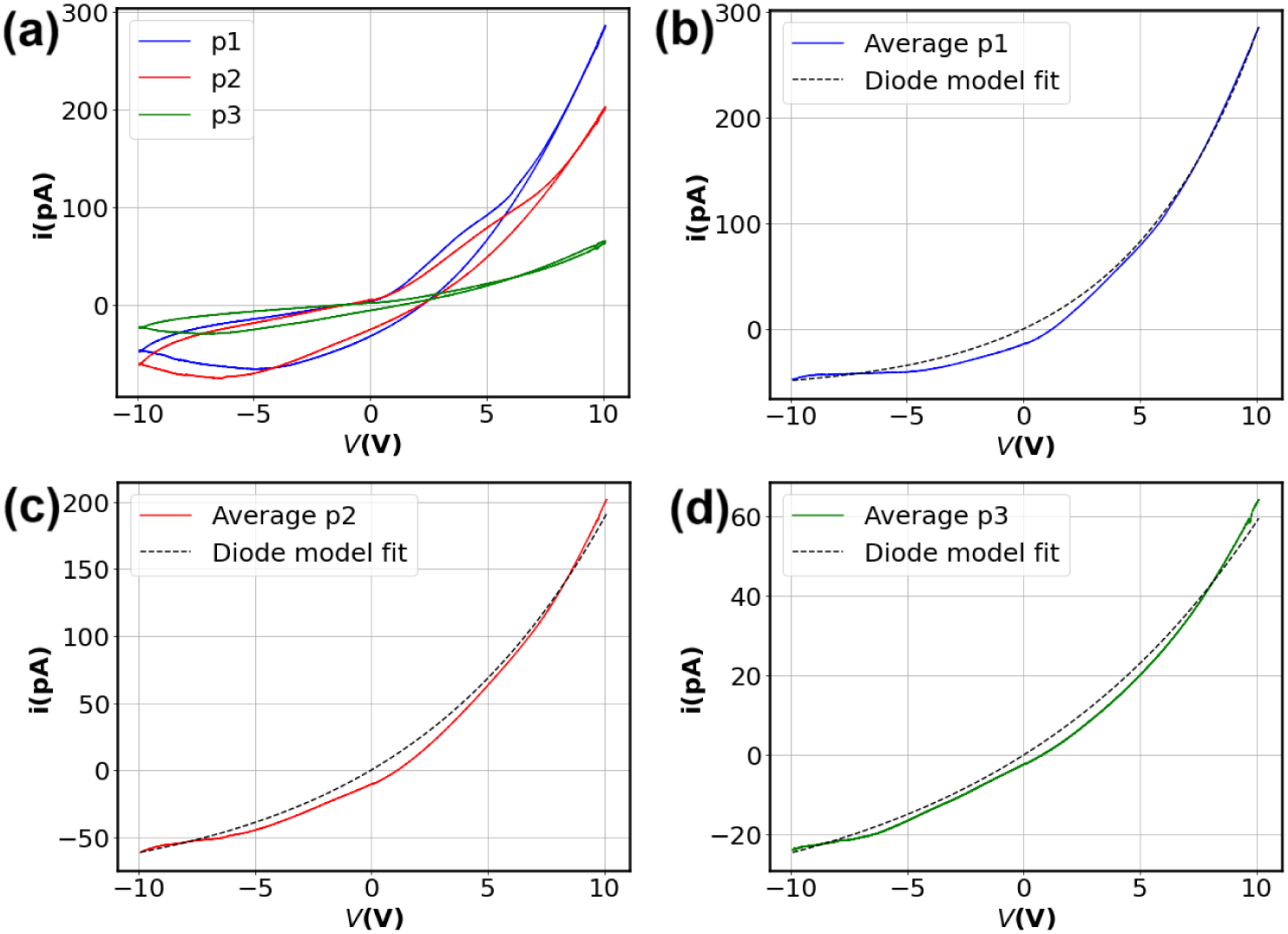
Current–voltage (I–V) characterization of OFCOLII osteoblast cells measured by CAFM. (a) Individual I–V curves acquired at the three locations indicated in Figure 5a (p1–p3) under a voltage sweep from −10 V to +10 V. (b–d) Averaged I–V responses at p1 (nucleolus), p2 (nucleus), and p3 (cell periphery), respectively. Dashed lines represent fits to a phenomenological diode-like model, highlighting pronounced nonlinear electrical responses that vary across different cellular regions.

As shown in Figure 6b–d, the averaged I–V curves display the highest current amplitudes at the nucleolus (p1) and the lowest at the cell periphery (p3). While the overall curve shape is qualitatively similar across the three regions, the degree of nonlinearity varies spatially, where capacitive displacement currents and field-dependent transport effects are expected to play a more prominent role. Parasitic capacitance contributions were corrected following the procedure described in ref 41. Due to the reduced signal-to-noise ratio at the peripheral region (p3), only high-quality curves with *R* ^2^ > 0.98 were retained for quantitative analysis.

To quantitatively capture the observed nonlinear electrical response, the averaged I–V curves were fitted using a phenomenological diode-like model,^50^

4 $$i=i_{0}\;\left(e^{aV}-1\right)$$

where *i* is the measured current, $i_{0}=\frac{nk_{B}T}{qR_{0}}$ , and $a=\frac{q}{nk_{B}T}$ . Here, *q* denotes the elementary charge, *k*_*B*_ the Boltzmann constant, *T* the absolute temperature, *n* the ideality factor, and *R*_0_ an effective resistance parameter. From this expression, an effective resistance parameter is obtained as $R_{0}=\frac{1}{i_{0}a}$ . The diode model is employed here as a phenomenological descriptor of the nonlinear electrical response and does not imply the formation of a classical semiconductor junction within the cell. Instead, the diode-like behavior should be interpreted as an effective voltage-dependent transport signature that likely reflects cell-type-dependent molecular and electrostatic heterogeneity. Such heterogeneity may introduce asymmetric interfacial barriers, local charge-trapping sites, and field-assisted transport pathways. Under an applied electric field, trapping and detrapping processes, together with interfacial polarization and barrier-limited transport, can produce rectification and the larger hysteresis observed in OFCOLII cells.

The resulting resistance distributions are shown in Figure 7. Mean resistance values of 119 GΩ, 158 GΩ, and 558 GΩ were obtained for points p1, p2, and p3, respectively. These results confirm a systematic increase in resistance from the nuclear region toward the cell periphery, consistent with the spatial organization of the cytoskeleton and the reduced electrical response observed in actin-rich regions. While impedance spectroscopy studies have previously characterized osteoblast monolayers^51,52^ the present results provide direct single-cell resistance measurements with subcellular spatial resolution.

**Figure 7:**
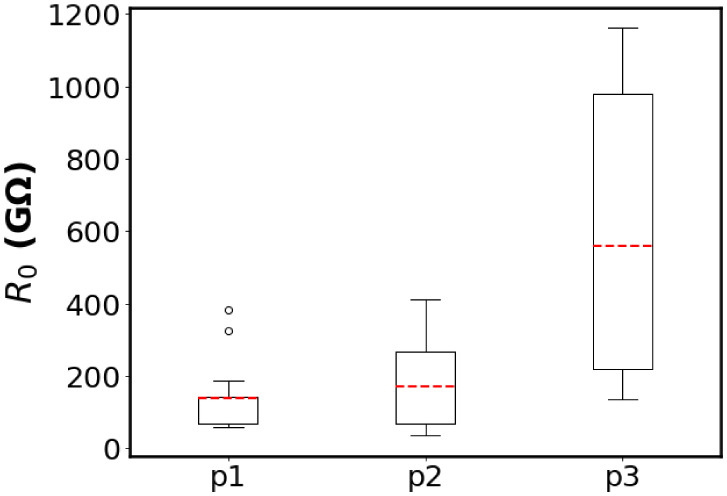
Comparison of effective electrical resistance extracted from CAFM measurements at different intracellular locations in OFCOLII osteoblast cells. Resistance values obtained at p1 (nucleolus), p2 (nucleus), and p3 (cell periphery) are shown (*n*_*p*1_ = 12, *n*_*p*2_ = 12, *n*_*p*3_ = 9). Boxes represent the interquartile range (Q1–Q3), whiskers extend to Q1 – 1.5 × IQR and Q3 + 1.5 × IQR, red dashed lines indicate mean values, and circles denote outliers. The data reveal a systematic increase in resistance from the nuclear region toward the cell periphery.

Notably, the extracted resistance parameter *R*_0_ increases as the local cell height decreases, indicating an inverse correlation between the effective electrical conductance and local cellular thickness. This trend suggests that nanoscale charge transport in OFCOLII cells is strongly modulated by local structural composition. In particular, regions enriched in actin filaments exhibit higher effective resistance, potentially reflecting the influence of filament density and orientation on charge propagation pathways.^53^ Although compositional heterogeneity likely contributes to the observed nonlinear response, further studies combining CAFM with compositional or perturbative assays will be necessary to isolate the dominant transport pathways. Furthermore, the diode-like behavior implies a voltage-dependent resistance that decreases exponentially with applied bias,^50^

5 $$R\left(V\right)=R_{0\;}e^{-aV}$$

indicating enhanced conductivity at higher positive voltages. Such behavior is consistent with field-assisted transport or barrier-lowering effects within the complex cellular matrix, rather than with purely ohmic conduction.

The present measurements were performed on fixed, air-dried cells to ensure mechanical stability and to suppress active ionic transport and electrochemical reactions during CAFM. Chemical fixation and drying are expected to modify the absolute magnitude of the measured electrical response, particularly by reducing ionic mobility, hydration-dependent conduction pathways, and active physiological currents. Therefore, the currents and resistances reported here should be interpreted as effective electrical responses of fixed cellular structures rather than as direct measurements of living-cell electrophysiology. Nevertheless, because all samples were prepared and measured under identical fixation, drying, substrate, probe, and CAFM measurement conditions, the relative spatial trends and the contrast between L929 fibroblasts and OFCOLII osteoblasts remain meaningful. In particular, the enhanced electrical response of nuclear regions relative to the cytoplasmic periphery and the distinct linear versus nonlinear I–V behavior of the two cell types reflect reproducible differences in cellular organization under the present experimental conditions. Future work comparing live, fixed, hydrated, and dried preparations will be important to determine how sample state modulates the CAFM electrical response.

## Conclusions

In this work, we established a reproducible conductive atomic force microscopy (CAFM) protocol to quantify local electrical transport in fixed single cells with subcellular resolution. Comparative measurements on L929 fibroblasts and OFCOLII osteoblasts revealed clear cell-type-dependent electrical responses: fibroblasts exhibited predominantly linear, ohmic-like I–V behavior with minor capacitive contributions, whereas osteoblasts displayed strongly nonlinear and hysteretic diode-like characteristics consistent with voltage-dependent transport and charge-storage effects. In both cell types, the nuclear region consistently showed higher currents and lower effective resistance than the cytoplasmic periphery, highlighting the role of intracellular organization in shaping the nanoscale electrical response. These results demonstrate that CAFM can resolve subcellular electrical heterogeneity and distinguish cellular phenotypes through label-free nanoscale transport signatures. The observed contrast between fibroblasts and osteoblasts also suggests that CAFM-based electrical measurements may provide a useful framework for future studies of altered or pathological cellular states, in which disease- or infection-induced changes in molecular organization, ion distribution, membrane integrity, hydration state, or cytoskeletal architecture could modify the local electrical response.

## Supplementary Material



## Notes

Figure 1SM: reproducibility of repeated CAFM I–V measurements in L929 fibroblast cells; Figure 2SM: reproducibility of repeated CAFM I–V measurements in OFCOLII osteoblast cells; Figure 3SM: CAFM maps acquired over selected 2 × 2 μm^2^ subcellular regions in an L929 fibroblast cell; Figure 4SM: CAFM maps acquired over selected 2×2 μm^2^ subcellular regions in an OFCOLII osteoblast cell (PDF)
