# Supplementary material for: Nonlinear Nanoscale Electrical Transport Distinguishes Fibroblasts and Osteoblasts
Source: ACS Omega. 2026 Jun 15;11(25):37560–9. doi: 10.1021/acsomega.6c02152 (PMC13325136; doi:10.1021/acsomega.6c02152)
Supplement: Supplementary file 1 [file ao6c02152_si_001.pdf]

Supplementary Material: Nonlinear nanoscale electrical transport distinguishes fibroblasts and osteoblasts

E. B. Torres,[1] R. Freire,[2] B. Sousa,[3] F. C. C. S. Salomão,[4] E. B. Barros,[1] J. S. de Sousa,[1] and C. L. N. Oliveira[1]

[1]*Departamento de Física, Universidade Federal do Ceará, Fortaleza, Ceará, Brazil*
[2]*Central Analítica, Universidade Federal do Ceará, Fortaleza, Ceará, Brazil*
[3]*Departamento de Bioquímica e Biologia Molecular, Universidade Federal do Ceará, Fortaleza, Ceará, Brazil*
[4]*Universidade Estadual do Ceará, Faculdade de Filosofia Dom Aureliano Matos, Limoeiro do Norte, Ceará, Brazil*

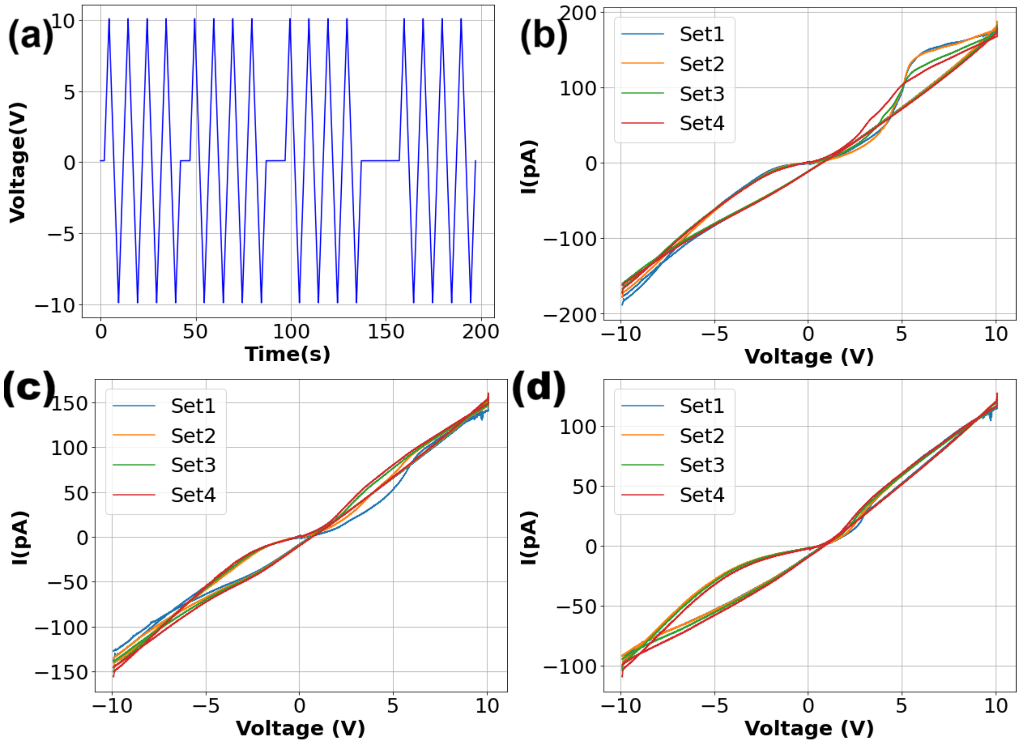

FIG. 1SM: Reproducibility of repeated CAFM I–V measurements in L929 fibroblast cells. (a) Voltage waveform applied during consecutive I–V acquisitions, showing repeated sweeps between −10 V and +10 V. (b–d) Four consecutive sets of I–V curves acquired at the same selected subcellular locations. The repeated measurements preserve the same approximately linear current–voltage behavior, with no significant signal drift, current loss, or progressive modification of the sample. These data confirm that the ohmic-like response observed in L929 cells is reproducible and not caused by tip wear or measurement-induced degradation.

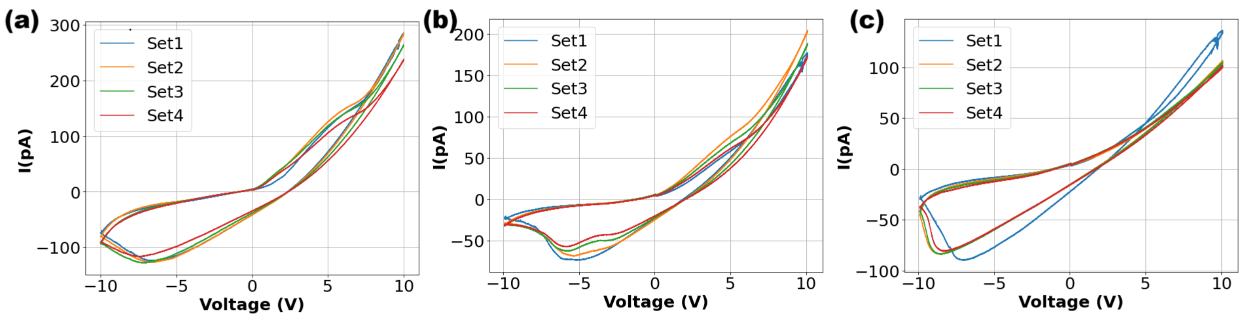

FIG. 2SM: Reproducibility of repeated CAFM I–V measurements in OFCOLII osteoblast cells. (a–c) Four consecutive sets of I–V curves acquired at the same selected subcellular locations. The nonlinear and hysteretic current–voltage response remains stable over repeated acquisitions, with no significant signal drift, current loss, or progressive modification of the sample. These data confirm that the diode-like response observed in OFCOLII cells is reproducible and does not arise from measurement-induced degradation during CAFM measurements.

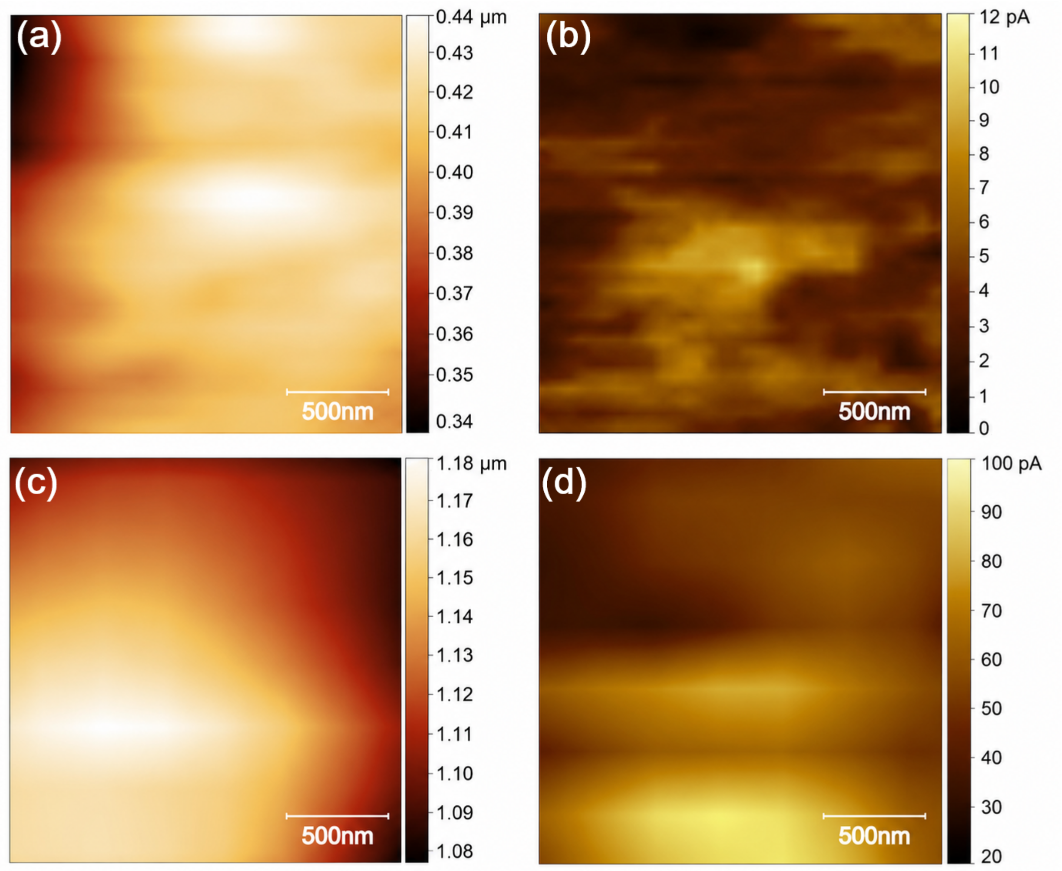

FIG. 3SM: CAFM maps acquired over selected 2 × 2 μm^2 subcellular regions in an L929 fibroblast cell. (a,b) Topographic image and corresponding current map acquired in the cell body/peripheral region. (c,d) Topographic image and corresponding current map acquired in the nuclear region. The maps reveal local heterogeneity in both topography and current distribution at the subcellular scale, further supporting the spatially resolved CAFM analysis presented in the main text. Scale bars: 500 nm.

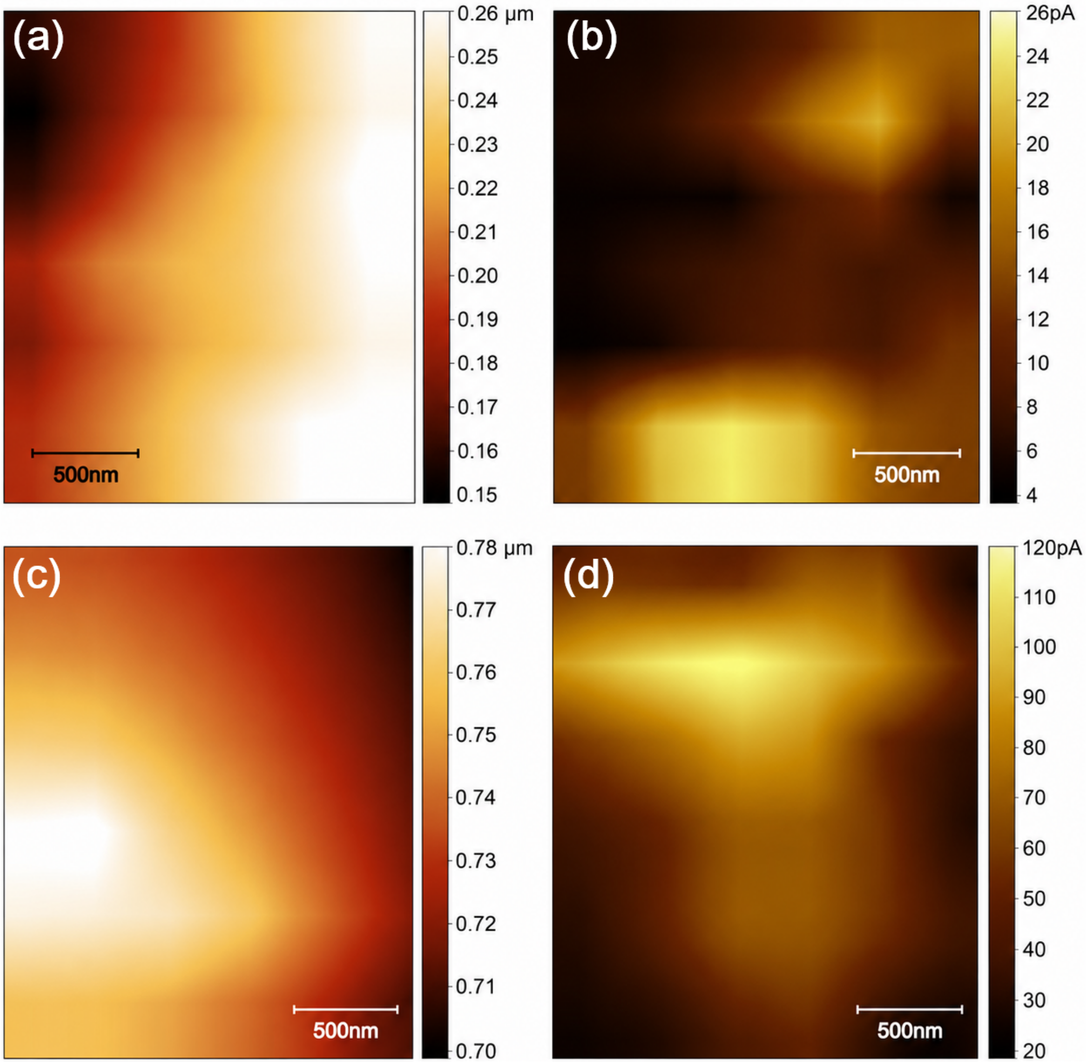

FIG. 4SM: CAFM maps acquired over selected 2×2 μm^2 subcellular regions in an OFCOLII osteoblast cell. (a,b) Topographic image and corresponding current map acquired in the cell body/peripheral region. (c,d) Topographic image and corresponding current map acquired in the nuclear region. The maps show nanoscale variations in topography and current response across selected subcellular regions, further supporting the spatial heterogeneity of the CAFM signal observed in the main measurements. Scale bars: 500 nm.
